# The chloroplast genome of *Sonneratia ovata*: genome structure and comparative analysis

**DOI:** 10.1080/23802359.2021.2008824

**Published:** 2022-01-30

**Authors:** Shi-Quan Wang, Feiyan Ren

**Affiliations:** Ministry of Education Key Laboratory for Ecology of Tropical Islands, Key Laboratory of Tropical Animal and Plant Ecology of Hainan Province, College of Life Sciences, Hainan Normal University, Haikou, China

**Keywords:** *Sonneratia ovata*, chloroplast, phylogenetic analysis, illumina

## Abstract

*Sonneratia ovata* is one of the most widely distributed mangrove species worldwide. In this study, the complete chloroplast (cp) genome of *S. ovata* was sequenced and assembled; its phylogenetic position was confirmed in Lythraceae. The total size of cp genome was 153,052 bp, exhibiting a typical quadripartite structure with a large single copy (LSC) region of 87,238 bp and a small single copy (SSC) region of 18,002 bp, two inverted repeats (IRs) regions of 23,906 bp each. The overall GC content was 37.3%, respectively. We detected 128 genes in cp genome, including 84 protein-coding genes, 36 tRNA genes, and 8 rRNA genes. A phylogenetic analysis showed that *S. ovata* has a close relationship with *S. apetala* within the genus *Sonneratia*.

*Sonneratia ovata* is one of the most widely distributed mangrove species worldwide. It belongs to the family Lythraceae, which is the most important producer in the ecosystem of the bay estuary and plays an important role in biodiversity conservation, environmental protection and ecotourism (Nehru and Balasubramanian 2012). However, there is no complete chloroplast of *S. ovata* report in NCBI. In this study, we sequenced the chloroplast of *S. ovata* and confirmed the phylogenetic position in family Lythraceae.

The leaf samples of *S. ovata* were collected from Wenchang, Hainan, China (19.6256840°N, 110.8144230°E). The voucher specimen (SO20200505) was deposited in the Laboratory of College of Life Sciences of Hainan Normal University, Haikou (Contact person: Shi-Quan Wang; Email: wsqmah@163.com). We used the fresh leaves to extract total genomic DNA with the modified CTAB method (Doyle and Doyle 1987) and constructed the libraries with an average length of 350 bp using the NexteraXT DNA Library Preparation Kit (Illumina, San Diego, CA), after that the libraries were sequenced on Illumina Novaseq 6000 platform, then 4.78 Gb clean data was assembled with *de novo* assembler SPAdes v.3.11.0 software (Bankevich et al. 2012). Finally, we annotated the complete cp genome by PGA software (Qu et al. 2019) with the CP genome *S. apetala* (MH986669) as reference, and submitted to GenBank under the accession number of MW266118.

The complete chloroplast genome of *S. ovata* was a circular double-stranded DNA molecule with the size 153,052 bp. Just like other angiosperms, the circular cp genome of *S. ovata* presented a typical quadripartite structure with a LSC (87,238 bp), a SSC (18,002 bp) and a pair of IR regions (IRa and IRb, each 23,906 bp). The total GC content of *S. ovata* cp genome was 37.3%.

We detected 128 genes in chloroplast genome of *S. ovata*, with 84 protein-coding genes, 36 tRNAs, and 8 rRNAs. There were 19 intron-containing genes in *S. ovata* cp genome, including 6 tRNA genes and 13 protein-coding genes. 15 genes comprised a single intron (*rpl*16*, rps*16*, rpo*C1*, trn*K-UUU*, trn*L-UAA*, ndh*A*, pet*B*, pet*D, *atp*F, 2 of *trn*I-GAU, 2 of *trn*A-UGC and 2 of *ndh*B,) and 4 genes (2 of *rps*12, *ycf*3 and *clp*P) had two introns.

We aligned the complete chloroplast genome of *S. ovata* with 12 species in Lythraceae by using Mafft v 7.309 (Katoh et al. 2002) with strategy of FFT-NS-2. Then we used model finder to select TVM + F+I + G4 model, and two taxa (*Lumnitzera racemosa* NC042408 and *Laguncularia racemosa* NC042719) from *Combretaceae* as outgroups, then used MEGA7 to construct maximum likelihood (ML) tree with 1,000 bootstrap. (Kumar et al. 2016). The phylogenetic tree showed that *S. ovata* was closely related to *S. apetala* (Figure 1). Meanwhile, *S. ovata* had a closest relationship with *S. apetala* in *Sonneratia*. The cp genome sequence of *S. ovata* in this study might provide useful information for Lythraceae plants researches.

**Figure 1.**
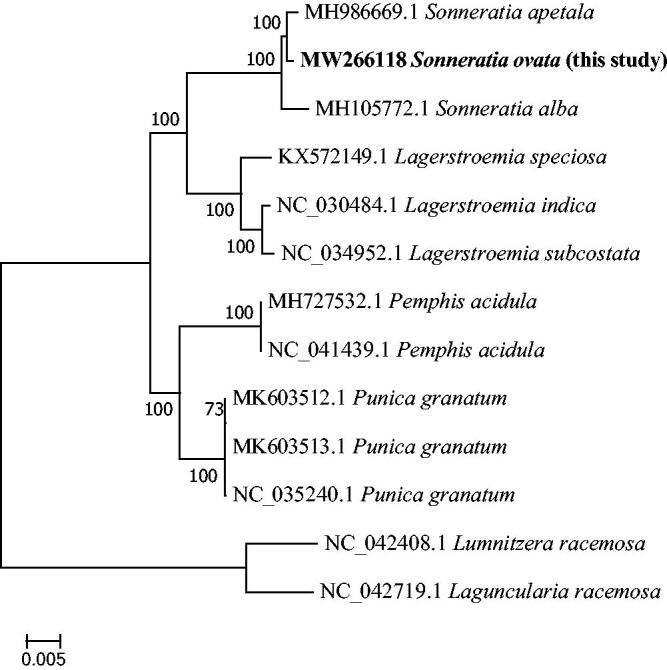
Maximum-likelihood phylogenetic tree for *S. ovata* based on 13 complete chloroplast genomes.

## Data Availability

The genome sequence data that support the findings of this study are openly available in GenBank of NCBI at (https://www.ncbi.nlm.nih.gov/) under the accession no. MW266118. The associated BioProject, SRA, and Bio-Sample numbers are PRJNA732004, SRR14623176, and SRS9041940, respectively.
